# Supplementary material for: Visceral Leishmaniasis-HIV Coinfection as a Predictor of Increased Leishmania Transmission at the Village Level in Bihar, India
Source: Front Cell Infect Microbiol. 2021 Mar 11;11:604117. doi: 10.3389/fcimb.2021.604117 (PMC7993201; doi:10.3389/fcimb.2021.604117)
Supplement: Supplementary file 1 [file Table_1.docx]

**SUPPLEMENTARY MATERIALS**

**Supplementary Table 1 :** Distribution of VL, VL-HIV, and PKDL cases per year (n (%)).

| **District** | **VL (%)** | **VL-HIV (%)** | **PKDL (%)** |
| --- | --- | --- | --- |
| **2014** | 889 (13·6) | 38 (9·6) | 17 (2·9) |
| **2015** | 706 (10·8) | 25 (6·3) | 29 (5·0) |
| **2016** | 476 (7·3) | 50 (12·6) | 33 (5·6) |
| **2017** | 1805 (27·7) | 124 (31·2) | 156 (26·7) |
| **2018** | 1306 (20·0) | 95 (23·9) | 161 (27·5) |
| **2019** | 1333 (20·5) | 65 (16·4) | 189 (32·3) |
| **Total** | **6515 (100.0)** | **397 (100.0)** | **585 (100.0)** |

**Supplementary Table 2 :** Distribution of VL, VL-HIV, and PKDL cases per district (n (%)).

| **District** | **VL (%)** | **VL-HIV (%)** | **PKDL (%)** |
| --- | --- | --- | --- |
| **Darbhanga** | 905 (13·9) | 36 (9·1) | 16 (2·7) |
| **Muzaffarpur** | 885 (13·6) | 77 (19·4) | 83 (14·2) |
| **Samastipur** | 910 (14·0) | 54 (13·6) | 49 (8·4) |
| **Saran** | 1832 (28·1) | 84 (21·2) | 207 (35·4) |
| **Siwan** | 864 (13·3) | 44 (11·1) | 151 (25·8) |
| **Vaishali** | 1119 (17·2) | 102 (25·7) | 79 (13·5) |
| **Total** | **6515 (100.0)** | **397 (100.0)** | **585 (100.0)** |

**Supplementary Table 3 :** Cumulative incidence (CI) of VL, VL-HIV, and PKDL per 10,000 population per year for all six selected districts combined.

| **Year** | **CI VL/10,000**  **pop** | **CI VL-HIV/10,000 pop** | **CI PKDL/10,000 pop** |
| --- | --- | --- | --- |
| **2014** | 0·42 | 0·02 | 0·01 |
| **2015** | 0·33 | 0·01 | 0·01 |
| **2016** | 0·21 | 0·02 | 0·01 |
| **2017** | 0·80 | 0·05 | 0·07 |
| **2018** | 0·56 | 0·04 | 0·07 |
| **2019** | 0·56 | 0·03 | 0·08 |
|  |  |  |  |

**Supplementary Table 4 :** Cumulative incidence (CI) of VL, VL-HIV, and PKDL per 10,000 population per district between 2014-2019.

| **District** | **CI VL/10,000 pop** | **CI VL_HIV/10,000 pop** | **CI PKDL/10,000 pop** |
| --- | --- | --- | --- |
| **Darbhanga** | 0·42 | 0·02 | 0·01 |
| **Muzaffarpur** | 0·33 | 0·03 | 0·03 |
| **Samastipur** | 0·35 | 0·02 | 0·02 |
| **Saran** | 0·82 | 0·04 | 0·09 |
| **Siwan** | 0·47 | 0·02 | 0·08 |
| **Vaishali** | 0·58 | 0·05 | 0·04 |
|  |  |  |  |

**Supplementary Table 5 :** Sensitivity analysis for the final multivariate negative binomial model, showing Incidence Rate Ratios for each of the different districts.

|  | **HIV-VL** | | | **PKDL** | | |
| --- | --- | --- | --- | --- | --- | --- |
|  | **IRR** | **95% CI** | **P-value** | **IRR** | **95% CI** | **P-value** |
| Darbhanga | 3·43 | (1·87 - 6·28) | <0·001 | 2·41 | (0·76 - 7·59) | 0·134 |
| Muzaffarpur | 2·50 | (1·17 - 5·32) | 0·018 | 2·45 | (1·10 - 5·43) | 0·028 |
| Samastipur | 2·43 | (1·53 - 3·85) | <0·001 | 1·79 | (1·02 - 3·14) | 0·043 |
| Saran | 2·03 | (1·45 - 2·85) | <0·001 | 1·62 | (1·23 - 2·14) | 0·001 |
| Siwan | 1·57 | (1·01 - 2·44) | 0·043 | 2·72 | (1·93 - 3·85) | <0·001 |
| Vaishali | 2·91 | (1·96 - 4·30) | <0·001 | 1·69 | (1·16 - 2·50) | 0·009 |
|  |  |  |  |  |  |  |
